# A Short Review of the Venoms and Toxins of Spider Wasps (Hymenoptera: Pompilidae)

**DOI:** 10.3390/toxins13110744

**Published:** 2021-10-21

**Authors:** Daniel Dashevsky, Juanita Rodriguez

**Affiliations:** Australian National Insect Collection, Commonwealth Science & Industry Research Organisation, Australian Capital Territory, Canberra 2601, Australia; Juanita.Rodriguez@csiro.au

**Keywords:** tarantula hawk, parasitoid, solitary wasps, pompilidotoxin, anoplin

## Abstract

Parasitoid wasps represent the plurality of venomous animals, but have received extremely little research in proportion to this taxonomic diversity. The lion’s share of investigation into insect venoms has focused on eusocial hymenopterans, but even this small sampling shows great promise for the development of new active substances. The family Pompilidae is known as the spider wasps because of their reproductive habits which include hunting for spiders, delivering a paralyzing sting, and entombing them in burrows with one of the wasp’s eggs to serve as food for the developing larva. The largest members of this family, especially the tarantula hawks of the genus *Pepsis*, have attained notoriety for their large size, dramatic coloration, long-term paralysis of their prey, and incredibly painful defensive stings. In this paper we review the existing research regarding the composition and function of pompilid venoms, discuss parallels from other venom literatures, identify possible avenues for the adaptation of pompilid toxins towards human purposes, and future directions of inquiry for the field.

## 1. Introduction

Venoms—toxic secretions from one organism that are introduced to the internals of another organism through a mechanical injury [1,2]—have evolved many times in the animal kingdom and several times in the insects alone [3,4,5,6]. Insects account for the majority of species of eukaryotic life on earth and among the insects, the most speciose order is likely Hymenoptera, which includes sawflies, wasps, bees, and ants [7,8,9]. Virtually all female hymenopterans are venomous and, as a result of their staggering diversity, they make up the plurality of venomous animals [8,9]. Most of this diversity within Hymenoptera is due to parasitic lineages which first arose in the Permian and have explosively diversified since [6,9,10,11,12]. While some of these species parasitize plants, most take advantage of arthropod hosts in some way for their larval development; this is usually fatal to the host and in that case the wasps are referred to as parasitoids rather than true parasites [10,11]. The study of insect venoms has accelerated recently for the purposes of evolutionary biology and the biodiscovery of nature-based laboratory tools and medicines [13,14,15]. However, parasitoid wasps have received relatively little attention, especially in proportion to the number of species.

These wasps attack a wide range of hosts and have evolved an almost equally broad array of specialized adaptations [16]. In some lepidopteran parasitoids of the family Encyrtidae (this phenomenon has been confirmed in the genera *Pentalitomastix* and *Copidosomopsis* and is likely even more widespread among other polyembryonic taxa), precocious defender morphs are the first larvae to emerge from the polyembryoic egg and spend their entire life patrolling the host and killing competitors rather than maturing [17,18]. Wasps in the families Braconidae and Ichneumonidae have independently incorporated functional polydnaviruses into their genomes [19,20,21,22,23] which are then produced in the calyx cells of the ovaries and injected into the host along with the wasp’s eggs [24]. These polydnaviruses then disrupt the host’s innate immune system and allow the eggs of the wasp to escape a death by encapsulation in host blood cells [25,26]. Members of these two families are also some of the most well studied examples of behavioural modulation of hosts by parasitoids. Braconids of the genera *Cotesia* [27,28], *Glyptapanteles* [29], and *Microplitis* [30,31] all have larvae that develop non-lethally within a lepidopteran host larva, emerge from within the host to pupate, and manipulate the host into standing guard to repel predators and hyperparasitoids (and in some cases reinforcing the parasitoid cocoons with its own silk) until it finally starves. Another braconid, *Dinocampus coccinellae* has a similar relationship with the ladybird beetle *Coleomegilla maculata* which actually carries the wasp cocoon and helps protect it from a range of predators [32,33]; this manipulation, it turns out, is the effect of a virus injected by the wasp, but an RNA virus called DcPV rather than a polydnavirus [34]. Ichneumonid wasps from the *Polysphincta* genus group in the subfamily Pimplinae cause their spider hosts to create unusual web that are shaped to the benefit of the parasitoid and the species involved have been the subject of consistent research efforts (reviewed in [33,35,36]). *Bassettia pallida*—a cynipid oak gall wasp which alters the host oaks’ morphology to provide small chambers, or crypts, in which the larvae develop—is itself victim to attack by *Euderus set*, a eulophid wasp [36]. Normally, *B. pallida* will bore holes from the crypt to the surface of the oak and fly away, but, when parasitized by *E. set*, they stay in the hole they bore and plug the entrance with their heads [37]. This maintains the crypt as a protected space for the *E. set* larvae to continue to feed on the body of *B. pallida*, but also allows them to escape when they are matured, since *E. set* can only bore their way out of a crypt with difficulty.

In Aculeata, the ovipositor is used only for stinging and, as a result, has adapted to become a specialized venom delivery mechanism [38]. While this comes at the cost of preventing aculeates from pursuing endoparasitic strategies, it allows the parasitoid lineages to subdue prey that is more active or better defended than the targets of non-aculeate parasitoids which virtually all attack soft or sedentary victims [39]. This is perhaps best exemplified by the emerald jewel wasp (*Ampulex compressa*), which is notorious for engaging in combat with much larger cockroaches and using precise stings first to the body of the prey to paralyze its legs and then to the brain which renders the cockroach either unwilling or unable to move of its own volition, but in a state that allows the wasp to lead it by the antennae to a burrow [40]. Detailed research into this species has found that they sting precise locations on their hosts to achieve particular neural manipulations such as the temporary paralysis of the legs, the aforementioned docility, and to stimulate the movement of the femur to allow the wasp access to the optimal location on the host leg upon which to lay her egg [41,42].

While the parasitoid wasps clearly use their venoms for parasitism through mechanisms such as paralysis, immune suppression, and developmental modulation, they can serve several other purposes as well including defense [43,44]. Several lineages of aculeates have convergently adopted eusocial lifestyles rather than the ancestral parasitoid strategy [12]. In these taxa, the venom system is used exclusively for defense of the colony and serves only as a deterrent with none of the other activities that are crucial to their parasitoid relatives [45]. To date, the majority of insect venom research has focused on eusocial hymenoptera. This literature is so extensive that only the aspects most directly relevant to the study of pompilid venoms will be mentioned [46,47,48,49]. While these venoms are extremely interesting and worthy of study, the multifunctional nature of parasitoid venoms alongside the sheer taxonomic diversity of parasitoid lineages suggests that they represent much richer prospects for discovering novel toxin activities or promising lead compounds for the design of new molecular tools or medications [50,51].

**Figure 1:**
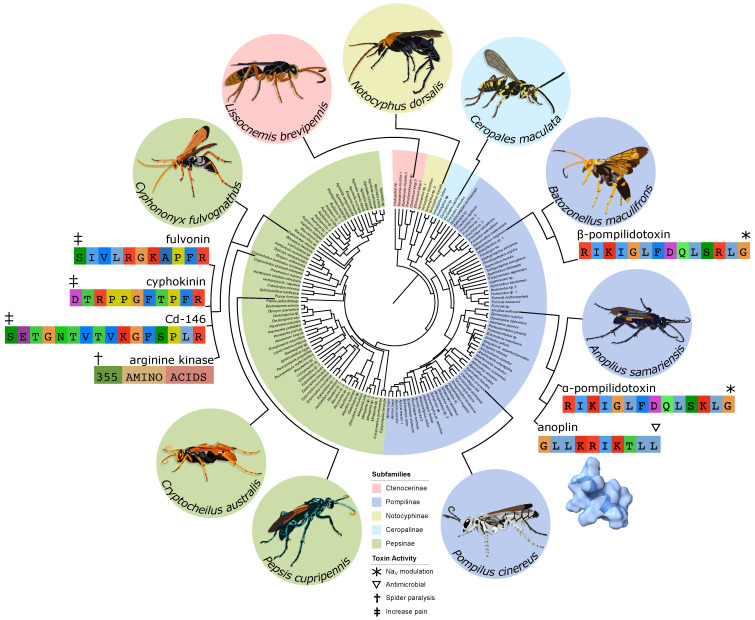
Phylogeny of Pompilidae with subfamilies highlighted and images to show species and toxins of interest (topology adapted from Waichert et al. [87]).

A family of spider specialist parasitoid wasps, Pompilidae (see Figure 1), has achieved a global distribution and plays an important role in most subarctic ecosystems [52,53,54,55,56,57,58,59,60]. These wasps not only have significant interactions with local spider fauna, but the adults largely subsist on nectar and thereby serve as pollinators as well [61,62,63,64,65,66,67,68,69]. Many of these wasps will feed at multiple flower species which pursue generalist pollination strategies [61,66,70,71,72], but a number of plants from Africa, Central and South America, and Australia have been documented to form specific pollination relationships with particular pompilid species [73,74,75,76,77,78,79,80,81]. Most of these systems appear to attract the wasps via scent signals [76,81] and some have specialized enough to make use of specific deceptive tactics by mimicking prey or mates [73,79]. The venoms of pompilids are notoriously painful [82] and the genus *Pepsis* is one of the few taxa that has been rated as a four (out of four) on the Schmidt Sting Pain Index (a subjective ranking of the pain caused by various insect stings) [83]. However, this defensive use is not the primary evolutionary purpose of these venoms. Stereotypically, pompilids reproduce by stinging a spider to paralyze it and dragging it across the ground (or the water in some exceptional cases [64]) to a burrow where an egg is deposited on it before being sealed in; when the larva hatches it consumes the paralyzed spider before pupating and emerging from the burrow as an adult [84,85,86].

## 2. Natural History Observations

Despite the fact that most pompilids are not host specialists (often choosing hosts based on size or ecology rather than specific taxa [88]), they still exhibit quite a bit of diversity in hunting tactics [85,89]. While some of these behavioral differences, such as what position the wasp carries the spider in or whether they dig their burrow before or after locating a host, are unlikely to significantly interact with the function of the venom, others may influence the selection pressures acting on the venom. This is perhaps easiest to imagine when we consider the duration of paralysis which, aside from pain, is probably the simplest metric available to characterize pompilid venoms. Rather than build a burrow, some pompilids allow the spider to continue living freely with the egg attached [90]. In this case it is likely to the wasp’s advantage for the host to recover quickly after oviposition. Other pompilids will amputate the legs of the spider after stinging it; not only does this make the spider easier to carry, but it likely makes it somewhat irrelevant whether the spider remains paralyzed in the burrow or not. And even amongst those pompilids that deposit whole spiders in their burrow, there can be variation between species in terms of how long it takes for the larva to fully develop. Paralysis lasting any longer than that would be evolutionarily neutral at best.

The rough duration of paralysis is the easiest metric for characterizing pompilid venoms and entomologists have recorded a number of observations. Interestingly, we observe clear taxonomic variation in the rough time scale reported (see Figure 1 and Table 1). Wasps of the genus *Anoplius* (subfamily Pompilinae) usually only produce paralysis lasting a matter of hours, while *Pompilus* (subfamily Pompilinae) tends towards several weeks, and large genera such as *Cryptocheilus* and *Pepsis* (subfamily Pepsinae) may immobilize their prey for months on end. Obviously not all paralyzed spiders survive these stings, indeed the tarantulas cared for by Costa et al. [91] must have been well fed beforehand to survive eight months of paralysis without starving. These observations suggest a phylogenetic pattern in the duration of paralysis, Pompiline (especially *Anoplius* species) wasps appear to paralyze their prey for much shorter time periods than Pepsine lineages.

One confounding factor to this potential pattern is the size of the wasps. Larger wasps possess larger venom glands and reservoir and usually yield more when provoked into stinging collection tubes for research purposes [pers. obs. DSD]. Experimental evidence has shown that the the length of paralysis produced by *Cyphononyx fulvognathus* venom in *Heteropoda* spiders was correlated with the amount of venom administered [92]. This suggests that larger species of wasp may produce longer paralysis in their targets simply by virtue of increased size. Other aspects of the venom such as the pain inducing toxins may also be dose dependent, since the stings of smaller wasps seem to be much less painful than the larger ones [pers. obs. DSD]. Recent studies of venoms from Mutillidae, which is the sister family to Pompilidae, showed that the toxins responsible for paralyzing arthropods also produced dose-dependent pain responses in mammals [93].

**Table 1:** Natural history observations of paralysis duration in spiders after pompilid stings.

Wasp	Spider	Behavioral Notes	Paralysis	Further Notes	Ref.
*Paracyphononyx africanus*	Lycosidae	Spiders live freely with egg attached	15 min		[90]
*Fabriogenia* sp.	Lycosidae	Wasp amputated spider’s legs	<1 day	palps responded to stimuli	per. obs.
*Anoplius nigerrimus*	Lycosidae	Spiders restrained in small cells	1–2 h		[94]
*Anoplius apiculatus autumnalis*	*Arctosa littoralis*	Spiders restrained in small cells	1–2 h		[95]
*Anoplius apiculatus autumnalis*	Lycosidae	Spiders restrained in small cells	1–2 h		[96]
*Anoplius apiculatus pretiosus*	Lycosidae	Spiders restrained in small cells	1–2 h		[96]
*Anoplius semirufus*	Lycosidae	Spiders restrained in small cells	1–2 h		[96]
*Anoplius semirufus*	various	Spiders restrained in small cells	1–4 h		[97]
*Anoplius marginalis*	Lycosidae	Spiders restrained in small cells	2 h		[98]
*Anoplius tenebrosus*	unspecified	Spiders restrained in small cells	4 h		[97]
*Anoplius semirufus*	*Trochosa avara*	Spiders restrained in small cells	6 h		[99]
*Anoplius apiculatus pretiosus*	*Arctosa littoralis*	Spiders restrained in small cells	7 h		[100]
*Pompilus scelestus*	*Geolycosa rafaelana*		>1 d	nesting takes up to a day	[98]
*Pompilus quinquenotatus*	*Larinioides cornutus*		30 d		[89]
*Sericopompilus apicalis*	unspecified		>46 d	spiders died	[96]
*Pompilus bigutattus*	*Metepeira labyrinthea*		62 d		[89]
*Pepsis marginata*	*Cyrtopholis portoricae*		2.5 mo		[101]
*Cryptocheilus affinis*	unspecified		>4 mo	spiders died	[102]
*Pepsis cupripennis*	unclear if *Acanthoscurria suina*, *Eupalaestrus weijenberghi*, or both		8 mo		[91]

## 3. Lab Results

The first forays into laboratory examination of pompilid venoms yielded correspondingly preliminary information. *Pepsis chrysothemis* venom was found to lack the activity of kinins, a family of short peptides whose presence or absence was occasionally cited as an informative character for resolving high-level relationships among aculeate Hymenoptera [103,104,105]. Additionally, *Pepsis pallidolimbata pallidolimbata* venom was determined to be almost entirely non-lethal in laboratory rodents [106].

Perhaps the best characterized toxins from pompilid venoms are α- and β-pompildotoxin (see Figure 1): linear peptides, 13 amino acids long, which differ from one another by a single residue and in their potency, but seem to act through the same biochemical mechanism and were isolated from the pompiline species *Anoplius samariensis* and *Batozonellus maculifrons* respectively [107,108,109]. They were first tested in lobster walking leg stretcher muscle/nerve preparations and were found to enhance the excitatory and inhibitory postsynaptic potentials as well as potentiating excitatory postsynaptic currents [107]. In these effects, β-pompildotoxin was found to be about five times more potent than α-pompildotoxin [108]. Further investigations found that these toxins cause long bursts of presynaptic activity which in turn potentiate the postsynaptic neurons in both the lobster preparation and in rat trigeminal neurons [110]. This accords with previous results which found pompilidotoxins would disrupt synchronized firing in rat cortical neurons and leave only a very low level of uncoordinated firing [111]. It appears that the stimulation of presynaptic neurons can lead to overexcitation and subsequent blockage of those cells [112]. More detailed mechanistic studies indicate that the pompilidotoxins achieve these effects by delaying the inactivation of voltage-gated sodium (Na_V_) channels [110].

A follow-up looking at different populations of hippocampal cells found that the effects of these toxins varied across different cell types, due to different distributions of Na_V_ subtypes [113]. This supposition was strengthened by a study using HEK cells (which do not normally express any Na_V_ channels) to recombinantly express the rat versions of either Na_V_1.2 (found in the central nervous system) or 1.5 (found in cardiac myocytes) [114]. Electrophysiological recordings from these cells confirmed that β-pompildotoxin is subtype selective: the toxin produced an effect in those cells expressing Na_V_1.2, but not those expressing Na_V_1.5. Expression of chimeric mutant versions of these channels further suggested that the extracellular linker between Segments 3 and 4 of Domain IV is the likely binding site for β-pompildotoxin [114].

To start to elucidate the molecular mechanisms of the toxins themselves, Konno et al. [115] created synthetic mutated analogues of α-pompildotoxin and found that the basic residues at positions 1, 3, and 12 were particularly important for the toxin’s function. Another study corroborated these results by comparing the pompilidotoxins to an unrelated anemone toxin (ATXII) which is also a linear peptide, possesses a similar pattern of basic residues, and produces similar effects [116]. A more recent study, in fact, tested several mutant versions and found that switching arginine for lysine or vice versa at the 1 and 3 sites in β-pompildotoxin or the 1 site in α-pompildotoxin had only minor effects [114]. This same study compared the effects of both α- and β-pompildotoxin on seven mammalian Na_V_ subtypes (1.1–1.7) expressed in HEK cells and one insect sodium channel (DmNa_V_1) expressed in *Xenopus* oocytes [114]. They found that these channels tended to respond in three ways to the pompilidotoxins. Cells expressing the insect Na_V_ and Na_V_1.6 exhibited a large increase in the steady state current which was then correlated with a decrease in the fast component of inactivation. Na_V_1.1, 1.2, 1.3, and 1.7 saw a large increase in the slow component of inactivation along with a decrease in the fast component and a small effect on the steady state current. Finally Na_V_1.4 and 1.5 did not respond at all to the toxins [114]. This subtype selectivity has allowed researchers to use pompilidotoxins as laboratory tools to examine neurochemical mechanisms such as the production of resurgent currents and subsequent rapid firing of Purkinje neurons [117]. In fact, researchers have built libraries of synthetic analogs to try to manufacture small peptides with subtype selectivity to further their investigations of these ion channels [118].

It remains somewhat unclear exactly how these in vitro results relate to the real world effects of pompilid venoms. The delayed inactivation of sodium channels seems to overexcite presynaptic neurons which can block their firing altogether relatively quickly [110]. This might disrupt the synchronized firing which is necessary for locomotion in the spiders they attack. While these toxins have not been tested on arachnids specifically, or their Na_V_ channels, these results are consistent across crustacean and insect channels and arachnid Na_V_1 channels are thought to be fairly similar [119]. Unlike vertebrates, invertebrates have not experienced the extensive duplication, subspecialization, and localization of Na_V_ subtypes [120]. Na_V_ channels are also associated in the transmission of pain and it is also possible that these toxins could play a part in the defensive role of the venom [121,122]. Perhaps they might even perform both functions like the peptide toxins studied in mutillid venoms [93]. However, the study on mutillid venoms found that they produced their painful and paralytic effects by targeting cell membranes rather than sodium channels [93].

Another toxin from *Anoplius samariensis* (the same species that α-pompildotoxin was discovered in) is known as anoplin (see Figure 1) and has been shown to form pores in membranes that are selectively permeable to cations [123,124]. This leads to antimicrobial activity which has spurred a wide ranging effort to characterize the structure-function relationship of this toxin and create optimized synthetic analogs in hopes of producing a novel therapeutic agents (e.g., [125,126,127,128,129,130,131,132]). This membrane-attacking activity seems to be quite general because anoplin has also demonstrated antifungal properties and the ability to induce the expression of defensive genes in plants [133]. Many painful toxins from across several domains of life make use of similar pore-forming mechanisms [93,134,135,136,137], so anoplin is another candidate for this role. However, it was a fairly minor component of the venom in which it was discovered [123]. That may still be sufficient for use as a defensive deterrent if it does in fact cause pain with high potency.

Another interesting line of research focused on the bradykinin-related peptides (see Figure 1) from the venom of *Cyphononyx fulvognathus*. Some of these toxins were able to bind to bradykinin receptors and, while not painful themselves, amplified the pain response in rats [138]. Perhaps there might be a synergistic effect between multiple toxins where one toxin is mildly painful but the bradykinin-related peptides amplify the pain signal and make it a much more effective deterrent. A similar defensive synergy was recently reported involving cytotoxic three-finger toxins and phospholipase A_2_s in the venom of spitting cobras [139]. Similar toxins in the venom of scoliid wasps were found to block the nicotinic acetylcholine receptors in insects which prevents synaptic transmission and would paralyze an insect, so perhaps they might play a similar role in pompilids as well [140].

The most promising lead for a paralytic toxin from a pompilid however, is an arginine kinase (see Figure 1) also from *Cyphononyx fulvognathus*. Assay guided fractionation led the researchers to this particuar toxin and they found that injecting recombinant versions of it would paralyze spiders [92]. They found that the duration of paralysis was dose dependent which is of interest to our earlier discussion of variation in paralysis duration among the family (See Section 2). This also accords with findings from *Anoplius samariensis* where ultrafiltration was used to separate the venom and found that the high molecular weight fraction would paralyze the spiders while the low molecular weight fraction did not [141].

A number of studies have produced sequences of toxins of unknown function including Cd-125, As-126, and Bm-10 which were all identified by mass spectrometry [142]. Elastase-like protein and Cyd25 were isolated from *Cyphononyx fulvognathus* as part of the same study that characterized the paralytic arginine kinase [138]. As-fr-19 had a similar cysteine pattern to some anemone toxins and dendrotoxins and was discovered in the same study that paralyzed spiders with ultrafiltrated *Anoplius samariensis* venom fractions [141]. A study of *Pepsis mexicana*, *Pepsis terminata*, and *Anoplius nigritus* used Edman degradation to discover the sequence of one peptide from each of the *Pepsis* species and detected hyaluronidase and proteolytic enzymatic activity in the venom of *P. mexicana* [143]. More modern techniques allow for the discovery of an even greater number of peptides. Mass spectrometry allows for the high-throughput de novo sequencing of peptides and the one such method was used to identify 20 novel peptides from the venom of *Pepsis decorata* [144]. These identified but mysterious toxins suggest just how much there is to learn still even about the pompilid venoms that have been directly researched, much less those that remain completely unexamined.

## 4. Discussion

The previous research on the venoms of pompilids offers tantalizing hints at what we may yet discover, but there is clearly much work yet to be done. For some time now the gold standard approach to determining venom composition has required a combination of mass spectrometric proteomics and venom gland transcriptomics [145,146]. None of the studies that have applied mass spectrometry to pompilid venoms have been able to make use of accompanying transcriptomes [142,144,147,148]. This is crucial because high-throughput peptide sequencing from mass spectra relies on databases of known proteins. However, with very few available protein sequences from Pompilidae, it is difficult for these algorithms to return accurate or complete results. A properly assembled and quality-controlled transcriptome from the same species (ideally the same individual) as the venom being analyzed gives much greater confidence that the proteomics results will be complete and accurate. In turn the proteomics help to validate which transcripts in the assembly are genuinely present in the venom gland and translated into proteins [145,146]. It is also important to consider the potential role of toxins that are not proteins or peptides. Other classes of molecules are known to be present in venoms, but research on these components tends to lag behind due to the added difficulty of isolation and characterization [149]. Nonetheless recent research into these non-proteinaceous toxins have yielded significant results in other taxa such as snakes and scorpions [150,151,152].

Performing these analyses for a wider range of species will give us a much better idea of the full composition of these venoms and form a basis from which to answer a broad range of questions about these venoms and their role in the ecology of the wasp as a whole. Until now, the only venoms to be studied have come from just four genera: *Anoplius*, *Batozonellus*, *Cyphononyx*, and *Pepsis* [92,107,108,123,138,141,143,144]. These do cover the two major subfamilies of Pompilidae—Pompilinae (*Anoplius* and *Batozonellus*) and Pepsinae (*Cyphononyx* and *Pepsis*)—but there are many smaller subfamilies and broad swathes of the phylogeny even within the main two that have not been studied at all (see Figure 1) [87,88,153].

The results from Picolo et al. [138] strongly suggest that an arginine kinase is the paralytic toxin in the venom of *Cyphononyx fulvognathus*, but it is unclear whether similar toxins even occur in the venoms of other pompilids much less whether they are also the primary paralytic toxins. Experiments with mutillid venoms indicate that small pore-forming toxins can act to paralyze arthropods [93], so anoplin may also play a part in this function. Furthermore, a number of other organisms whose venom rapidly paralyzes their prey have convergently evolved toxins which delay the inactivation of Na_V_ channels including jellyfish, sea anemones, scorpions, spiders, cone snails, and snakes [154,155,156,157,158,159,160,161,162,163,164,165,166]; this suggests then that pompilidotoxins could potentially act as paralytic agents as well. Currently, the arginine kinase is the only paralytic toxin with direct evidence to back it up, but whether these other toxin classes may act synergistically or on different time scales needs to be investigated to fully explain the long-term paralysis produced by some pompilid species.

Other functions of the venom remain even more enigmatic. Anoplin may well cause pain through generalized pore-forming mechanisms and bradykinin related peptides may synergistically increase the perception of pain, but these are only hypotheses suggested by previous results and direct evidence will be needed before anything can be concluded about what makes these venoms so extremely painful. Other possible functions including preservation of the immobilized spider (perhaps mediated by the antimicrobial activity of peptides like anoplin [123]) or predigestion of the host for ease of consumption by the larva (as suggested by the proteolytic activity in *Pepsis mexicana* venom [143]) are almost entirely speculative at this point and might remain so without further behavioural and biochemical experiments.

Another worthy avenue of inquiry would be testing for differential venom composition. Previous results show that cone snails [167] and assassin bugs [168] can produce strikingly different venom composition in different situations. While the relatively simple reservoir anatomy of hymenopterans might make the possibility seem *a priori* less likely [106,169,170,171], testing for this phenomenon should be part of the due diligence when examining the venom system of any group of animals in-depth for the first time.

Genomic data will hopefully shed light on questions about these venoms in the future. While transcriptomic data can give the complete sequences of toxins, genomic data can provide much more information including intronic sequences, promoter regions, pseudogenes, chromosomal location, or syntenic patterns. The relations of small peptides such as pompilidotoxins, anoplin, or bradykinin-related peptides may prove to be almost impossible to unravel without this additional information because of their extremely small size; it is too easy for mutations to hit saturation in such a small target and repeatedly overwrite any phylogenetic signal. It has been suggested that many of these hymenopteran peptides form a toxin superfamily [172] and genomic data from a wider range of taxa may help confirm or deny that hypothesis. Comparative genomics also offers our best window into the evolutionary history of toxin recruitment which is an open research question even in the most thoroughly studied venomous taxa [173]. Experiments on the pteromalid wasp genus *Nasonia* suggested that many venom genes were the result of cooption of single genes [174]. This stands in contrast to popular intuition which suggested that most toxin recruitment would involve gene duplication of some sort in processes like subfunctionalization and neofunctionalization [5].

On top of questions of venom evolution in general, pompilid venoms may also lend insight to the evolution of Pompillidae as a family. Recruitment of particular toxins, expansion of toxin families, or crucial mutations could all suggest shifts in ecology or lend support to previously tenuous relationships. These wasps are highly prevalent pollinators and are intimately connected with spiders who are themselves crucial nodes in virtually every terrestrial food web. Understanding the ecology of Pompilids as both pollinator and parasite will likely be a facet of any future wholistic understanding of ecosystems on any continent except Antarctica.

The study of pompilid venoms contains the potential for a number of practical benefits as well. A neurotoxin from a funnel web spider (*Hadronyche versuta*) has been commercialized as an insecticide [175,176]. Venom peptide toxins make promising candidates for this application because the proteins are biodegradable and many are already evolved to be highly potent and specific. Many spider toxins are attractive because of their chemical stability due to a wide range of cystine-cystine bond patterns and because many spiders prey on insects which represent a large share of crop pests [177,178]. While many hymenopteran toxins, including the most well-known components of pompilid venoms, tend not to be stabilized by cystine-cystine bonds, this is far from a universal rule and others may yet be discovered. Additionally, pompilid venoms are specifically evolved to target arachnids: a number of mite species are significant pests and they are known to evolve resistance to previous pesticides relatively quickly [179,180,181,182,183,184]. Venoms have also been the source of a number of medications, most spectacularly snake coagulotoxins, which have been adapted or mimicked for a range of applications that include blood pressure medications, surgical anti-clotting drugs, and heart attack prevention [15,185,186,187,188,189,190,191,192,193]. Toxins from other organisms that have seen similar medicinal use include a cone snail-derived painkiller, a diabetes ameliorating drug from the Gila monster (*Heloderma suspectum*), and anticoagulants from the medicinal leech (*Hirudo medicinalis*) [15,194,195,196,197].

By far the best studied insect venom is that of the honeybee (*Apis mellifera*) [46,47,48,49] and in a purified form is an FDA-approved product for several painful inflammatory conditions [198,199]. Several systematic reviews have confirmed that *A. mellifera* venom can help alleviate pain in these conditions, but it remains unclear exactly which venom components are responsible for this effect or what mechanisms [200,201]. With venoms as poorly understood as those of pompilids it is hard to guess exactly where we might find benefit from them. In terms of the better known pompilid toxins, the pompilidotoxins join a range of other Na_V_-modulatory toxins which may one day be put to use or adapted to combat pain [121,122]. In the meantime they have already been used by neuroscientists to probe the distribution of some of the Na_V_ subtypes in mammals [117,118]. However, it is anoplin’s antimicrobial activity that has generated the most interest from researchers. Many avenues have been explored to try to optimize its medicinal properties [125,126,127,128,129,130,131,132]. Many other toxins and chemicals use similar pore-forming mechanisms as anoplin and have been pursued for their ability to kill cells [202,203]. In fact some current chemotherapy drugs work through a similar mechanisms and it is thought that proper targeting of pore-forming toxins could present a new opportunity for selective cell death in cancer and other diseases [204,205,206]. Two *A. mellifera* toxins in particular, mellitin and mastoparan have been rather heavily studied for these and other effects and mastoparan in particular is similar to anoplin in that both are short helical transmembrane peptides [199].

While there are no guaranteed outcomes in evolutionary biology or biodiscovery, it is clear that there is a vast wealth of knowledge and undiscovered molecules in the venoms of pompilids and other parasitoid wasps. The results summarized in this review represent only a hint of all there is to learn and gain from studying these systems.

## Abbreviations

The following abbreviations are used in this manuscript:

PMTX	pompilidotoxin
Na_V_	voltage-gated sodium channel
CSIRO	Commonwealth Science & Industry Research Organisation
CERC	CSIRO Early Research Career
